# Thermo-Regulation of Genes Mediating Motility and Plant Interactions in *Pseudomonas syringae*

**DOI:** 10.1371/journal.pone.0059850

**Published:** 2013-03-19

**Authors:** Kevin L. Hockett, Adrien Y. Burch, Steven E. Lindow

**Affiliations:** Department of Plant and Microbial Biology, University of California, Berkeley, California, United States of America; Virginia Tech, United States of America

## Abstract

*Pseudomonas syringae* is an important phyllosphere colonist that utilizes flagellum-mediated motility both as a means to explore leaf surfaces, as well as to invade into leaf interiors, where it survives as a pathogen. We found that multiple forms of flagellum-mediated motility are thermo-suppressed, including swarming and swimming motility. Suppression of swarming motility occurs between 28° and 30°C, which coincides with the optimal growth temperature of *P. syringae*. Both *fliC* (encoding flagellin) and *syfA* (encoding a non-ribosomal peptide synthetase involved in syringafactin biosynthesis) were suppressed with increasing temperature. RNA-seq revealed 1440 genes of the *P. syringae* genome are temperature sensitive in expression. Genes involved in polysaccharide synthesis and regulation, phage and IS elements, type VI secretion, chemosensing and chemotaxis, translation, flagellar synthesis and motility, and phytotoxin synthesis and transport were generally repressed at 30°C, while genes involved in transcriptional regulation, quaternary ammonium compound metabolism and transport, chaperone/heat shock proteins, and hypothetical genes were generally induced at 30°C. Deletion of *flgM*, a key regulator in the transition from class III to class IV gene expression, led to elevated and constitutive expression of *fliC* regardless of temperature, but did not affect thermo-regulation of *syfA*. This work highlights the importance of temperature in the biology of *P. syringae*, as many genes encoding traits important for plant-microbe interactions were thermo-regulated.

## Introduction

The plant pathogen *Pseudomonas syringae* has been studied extensively both as a saprophyte, living epiphytically on plant surfaces as well as a pathogen residing within the leaf apoplast. The epiphytic phase is an important part of the disease cycle for these pathogens since it provides inoculum for subsequent infection. Invasion of plants is apparently a relatively rare event, necessitating an abundant epiphytic population on leaves for disease to be likely to occur [1]. Thus most cells occur as epiphytes prior to invasion into the leaf interior. While an understanding of the biology of these pathogenic bacteria on leaf surfaces is of intrinsic scientific interest, it is also of practical significance since the traits that enable them to thrive as epiphytes contribute to diseases of important crop plants.

Leaf surfaces can be relatively hospitable under certain circumstances, but often provide a stressful habitat to bacteria due to conditions of periodic desiccation, UV irradiation, and nutrient limitation [2], [3]. Furthermore, the conditions on leaves are highly temporally variable, quickly switching between hospitable and inhospitable [2], [3]. Under stressful conditions (high irradiance and low relative humidity), the leaf surface, on balance, is inhospitable. However, some anatomical sites, such as the base of glandular trichomes or epidermal cell junctions, appear to offer protection against environmental extremes and are preferentially colonized by epiphytic bacteria [4]. These sites, often termed “preferred sites”, are likely sources of both water and nutrients [5].

Flagellar-mediated motility is an important trait for both the epiphytic and pathogenic lifestyles of *P. syringae* [6], [7], [8], [9]. Non-motile *P. syringae* mutants are more sensitive to desiccation and UV exposure than their motile counterparts presumably because they cannot access sites in which they can escape environmental stresses [6]. Additionally, non-motile mutants are severely reduced in their ability to invade the leaf interior or cause disease after topical application to plants [8], [9], [10]. Although motility is apparently beneficial for phyllosphere bacteria, such benefits may be conditional since motility would be dependent on the presence of at least some free water on plants to enable flagellar function [11]. The flagellum is a particularly costly organelle to synthesize, estimated to consume 2% of the cell’s biosynthetic energy expenditure in *E. coli* [12], so its expression is most likely highly suppressed under conditions that do not permit motility. Additionally, in many bacteria, including Pseudomonads, flagellum synthesis is suppressed when other traits such as the type III secretion system (T3SS) [9], [13], exopolysaccharide (EPS) production [9], [14], or biofilm formation [15] are expressed; all of these traits are critical for plant-pathogen interactions [16], [17].

Given the temporally and spatially variable nature of the leaf habitat, the conditional benefit of flagellar-mediated motility, and the inverse pattern of regulation of flagellum expression and other plant colonization traits, motility is likely regulated in a way that will maximize the fitness of *P. syringae* on leaves. By understanding the environmental parameters that modulate motility in *P. syringae*, we may gain insight into the conditions that promote invasion by this organism, as well as a sense of how different fitness factors are coordinately regulated under various environmental circumstances. In temperate regions, temperature may be a critical parameter influencing motility because it is a correlate with wetness of leaves. Leaves are unlikely to remain wet for lengthy periods under warm temperatures since this typically occurs during the day. In contrast, leaf wetness from either dew or rain is most likely to persist under cool temperatures that occur at night. Cells might therefore use temperature as a surrogate for leaf wetness and perhaps exhibit anticipatory behaviors that would maximize their fitness. Temperature is known to regulate certain virulence factors such as phaseolotoxin production in *P. syringae* pv. phaseolicola or coronatine in *P. syringae* pv. glycinea, reviewed in [18]. However, the more general role of temperature as a stimulus for changes in gene expression in *P. syringae* has not been well examined. Insights gained from an understanding of the temperature stimulon should illuminate the interactions of this pathogen with host plants.

In this report we investigate the effect of incubation temperature on global gene expression in *P. syringae*. Transcriptome analysis revealed that many genes known to be involved in plant colonization are temperature regulated. Notable among the genes affected by temperature were those involved in motility, including synthesis of the flagellum and syringafactin, a major surfactant produced by *P. syringae*.

## Results

### Swimming and Swarming Motility are Inhibited at Elevated Incubation Temperatures in *P. syringae*

**Figure 1:**
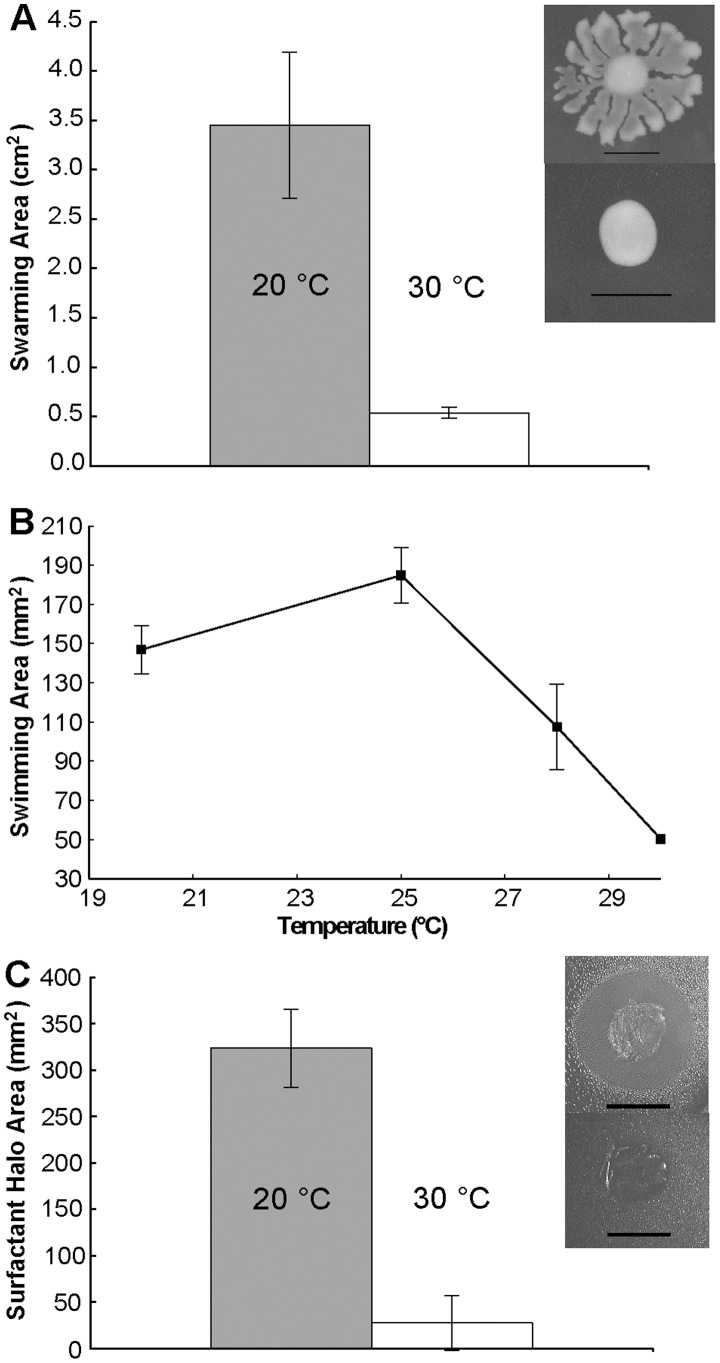
Thermo-regulation of motility in *P. syringae*. Swarming of *Pseudomonas syringae* B728a at 20°C (gray bar, top photo of inset) and 30°C (white bar, bottom photo of inset) after 24 hours (A). Area of swimming colonies of *Pseudomonas syringae* B728a after 24 hours growth at various temperatures (B). Biosurfactant area on plates following 24 hours of incubation at 20°C (gray bar) or 30°C (white bar) (C). The vertical bars represent the standard deviation of the mean.

Swarming motility of *P. syringae* on the surface of low-agar plates was much greater in cells incubated at 20°C than at temperatures of 28–30°C (Fig. 1A). As swarming motility in most bacteria involves some form of surfactant production and requires a functional flagellum [19], we assayed swimming motility of *P. syringae* through soft agar (which is flagellum-dependent) as well as the abundance of surfactants produced by *P. syringae* at 20°C and 30°C using the atomized oil assay [20]. Both the rate of swimming and the amount of surfactant produced by *P. syringae* were significantly reduced at 30°C compared to 20°C (Fig. 1B and C), suggesting that biosurfactant production as well as flagellum synthesis or function was suppressed at the warmer temperature.

### Syringafactin and Flagellin Expression are Repressed at Elevated Temperatures

**Figure 2:**
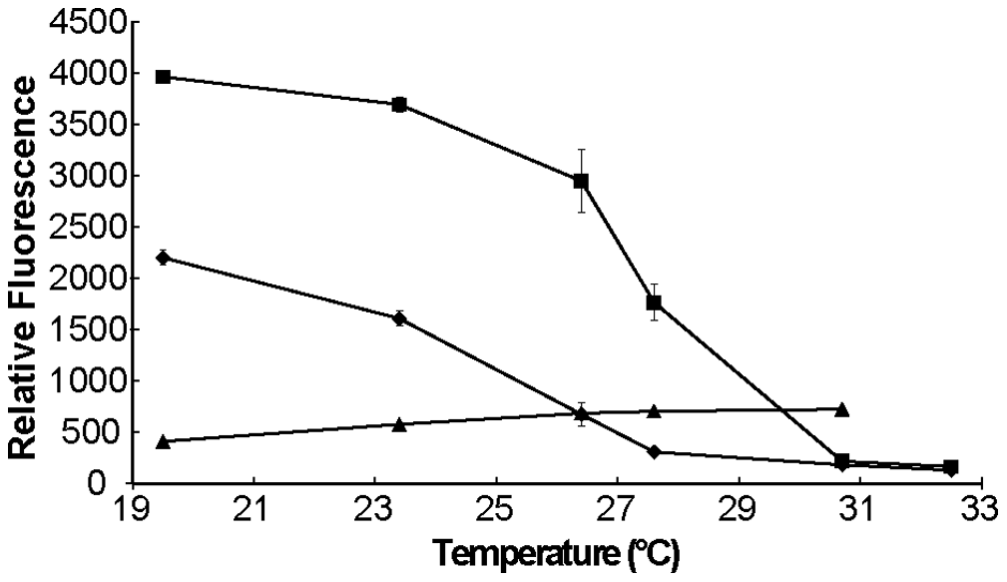
Thermo-regulation of *fliC* and *syfA* expression. Cell normalized GFP fluorescence of cells grown for 48 hours at various temperatures that harbored a fusion with promoters of *fliC* (square), *syfA* (diamond), or *nptII* (triangle). The vertical bars represent the standard deviation of the mean.

**Figure 3:**
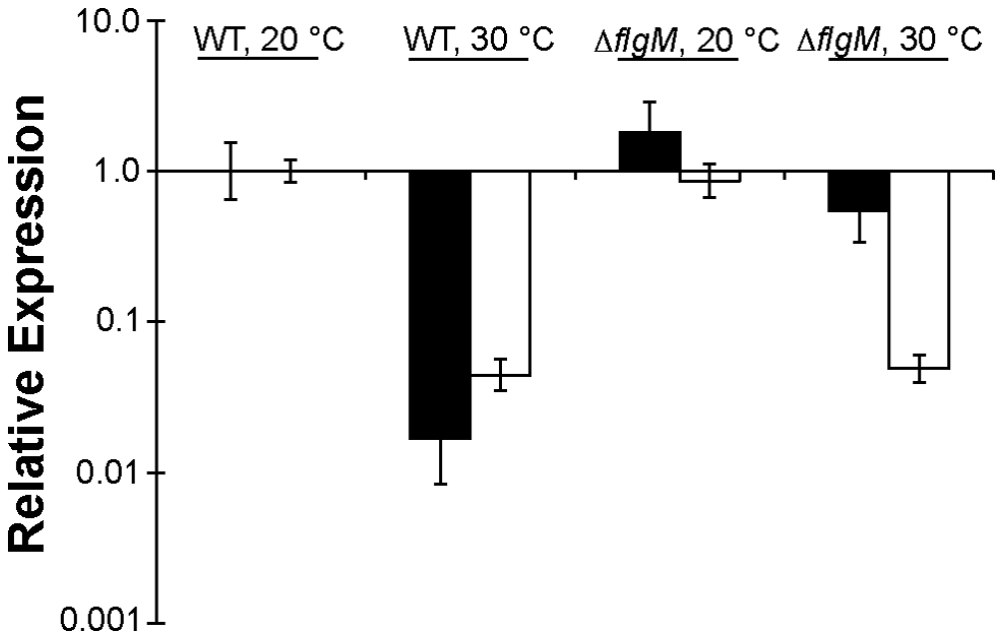
Lack of thermo-regulation of *fliC* in a Δ*flgM* mutant. Relative expression of either *fliC* (black bars) or *syfA* (white bars) in wild type *Pseudomonas syringae* B728a or a Δ*flgM* mutant at either 20°C or 30°C. Expression normalized to WT, 20°C. Error bars represent the 95% confidence interval.

Since swarming motility was strongly temperature-dependent, and temperature appeared to affect both flagellum and surfactant production, we investigated the influence of temperature on expression of genes associated with each trait. We measured the temperature-dependent expression of *fliC*, which encodes flagellin, and *syfA*, which encodes a non-ribosomal polypeptide synthase (NRPS) required for syringafactin (the major biosurfactant produced by *P. syringae* B728a) synthesis [20], [21], using both GFP reporter gene fusions and measurement of RNA abundance using qRT-PCR. The expression of *syfA* and *fliC* decreased greatly in the temperature range of 25 to 28°C (Fig. 2). While *syfA* is nearly fully repressed at 28°C the expression of *fliC* is only repressed by about 50% compared to its maximum expression at 19–20°C, but becomes nearly fully repressed at 30°C. The transcript abundances of both *syfA* and *fliC* as measured by qRT-PCR were also significantly lower in cells grown at 30°C compared to 20°C (Fig. 3).

### Thermo-regulation of the Flagellum Predominately Affects Stage III and IV Flagellar Genes

Using an RNA sequencing approach, we determined the global effect of temperature on the *P. syringae* transcriptome (see below for full description). We were particularly interested in assessing the effect of temperature on the flagellar cascade to determine if all genes in the cascade were thermo-regulated similar to *fliC*.

Assuming that regulation of *P. syringae* flagellum synthesis follows the same four-tier system of *Pseudomonas aeruginosa* [22], we found that thermo-regulation appeared to be prominent in class III genes, and to an even greater extent in class IV genes (Table 1). Analysis of the transcriptome suggested that the late stage (class III and IV) genes were the most strongly temperature-regulated genes in the flagellar cascade, with *fliC* being the most differentially expressed. Neither of the class I genes (*fleQ* and *fliA*) were temperature regulated. Interestingly, *morA*, a protein involved in c-di-GMP metabolism [23] (possessing both GGDEF and EAL domains) was the only gene directly involved in flagellar motility that was more highly expressed at 30°C.

**Table 1:** Thermo-regulation of flagellar genes.

	Class I	Class II	Class III	Class IV
number ofthermo-responsivegenes^a^	0/2	3/22	6/12	7/13
mean foldtemperatureeffect^b^	n/a	2.6	2.1	4.8

a Genes thermo-regulated within class/total number of genes within class.

b Determined only for the genes within class that are thermo-regulated.

### *flgM* is Required for Thermo-repression of *fliC* but not *syfA*

**Figure 4:**
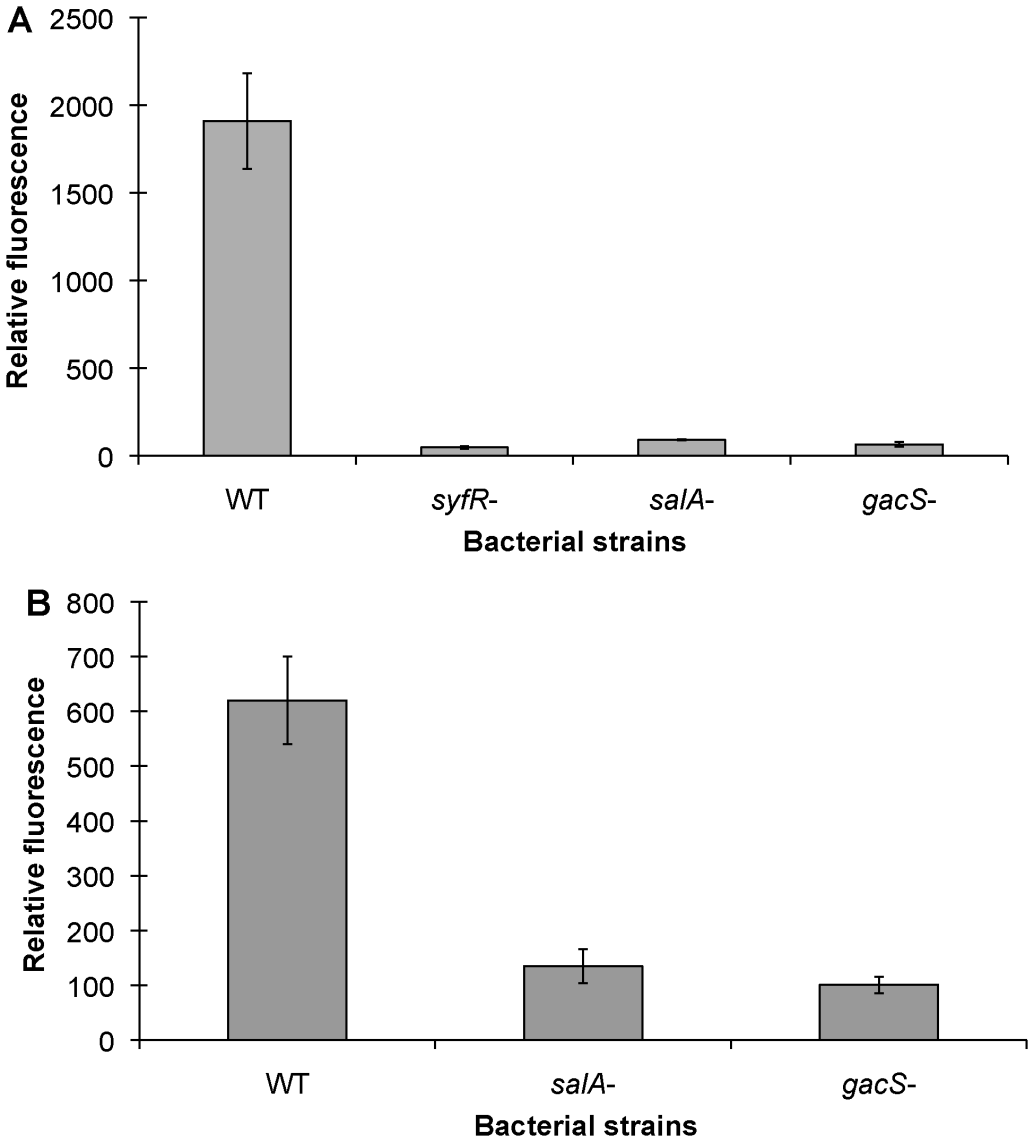
Reduction of *fliC* expression in Δ*flgM* by complementation. Cell-normalized GFP fluorescence of either wild type *Pseudomonas syringae* B728a (black squares) or a Δ*flgM* mutant (open diamonds) harboring a *gfp* reporter gene fusion with a promoter of *fliC* (A). Cell normalized GFP fluorescence of wild type *P. syringae* or a Δ*flgM* mutant harboring a *gfp* reporter gene fusion with the promoter of *fliC* as well as either a *flgM* complementing vector (p519Mcomp) (black and dark gray bar, respectively) or vector control (p519empty) (white and light gray bar, respectively) when grown at either 20°C or 30°C (B). The vertical bars represent the standard deviation of the mean.

The cascade of flagellum regulation has been elucidated in detail in both *Salmonella typhimurium* and *E. coli*, where the interaction of FlgM (an antisigma factor) and FliA (an alternative, flagellum-specific sigma factor) is required for the inhibition of expression of late stage genes (genes encoding the flagellar filament, cap, motor proteins, and chemotaxis components) until the hook and basal body has been completed [24]. Since analysis of the flagellar cascade revealed that the class IV flagellar genes were the most strongly affected by incubation temperature (*fliC* having the greatest difference in expression between warm and cool growth conditions), we hypothesized that the FlgM-FliA interaction might also mediate thermo-repression of class IV genes, and in particular, *fliC* expression in *P. syringae*. We compared *fliC* expression in a *flgM* deletion strain (Δ*flgM*) to that in the WT strain at both 20°C and 30°C. While the expression of *fliC* decreased with increasing growth temperature above 25°C in WT, its expression in Δ*flgM* was high and independent of growth temperature (Fig. 4A). Over-expression of *flgM in trans* suppressed the expression of *fliC* at both cool and warm temperatures in both the WT and Δ*flgM* strains (Fig. 4B). The transcript abundance of *fliC* measured by qRT-PCR also was much higher in Δ*flgM* compared to WT when cells were grown at 30°C (Fig. 3). These results indicate that *flgM* is required for thermo-repression of *fliC* and that its over-expression on a high copy number plasmid suppresses *fliC* at all temperatures.

To test whether *flgM* was also responsible for thermo-repression of *syfA* at 30°C, we assessed *syfA* expression in Δ*flgM* at 20°C and 30°C using qRT-PCR. Δ*flgM* exhibited thermo-repression of *syfA* similar to WT (Fig. 3). Additionally, Δ*flgM* produced less surfactant at 30°C than at 20°C, similar to WT (Fig. S1).

### *salA* Positively Regulates *syfR* and *syfA*

**Figure 5:**
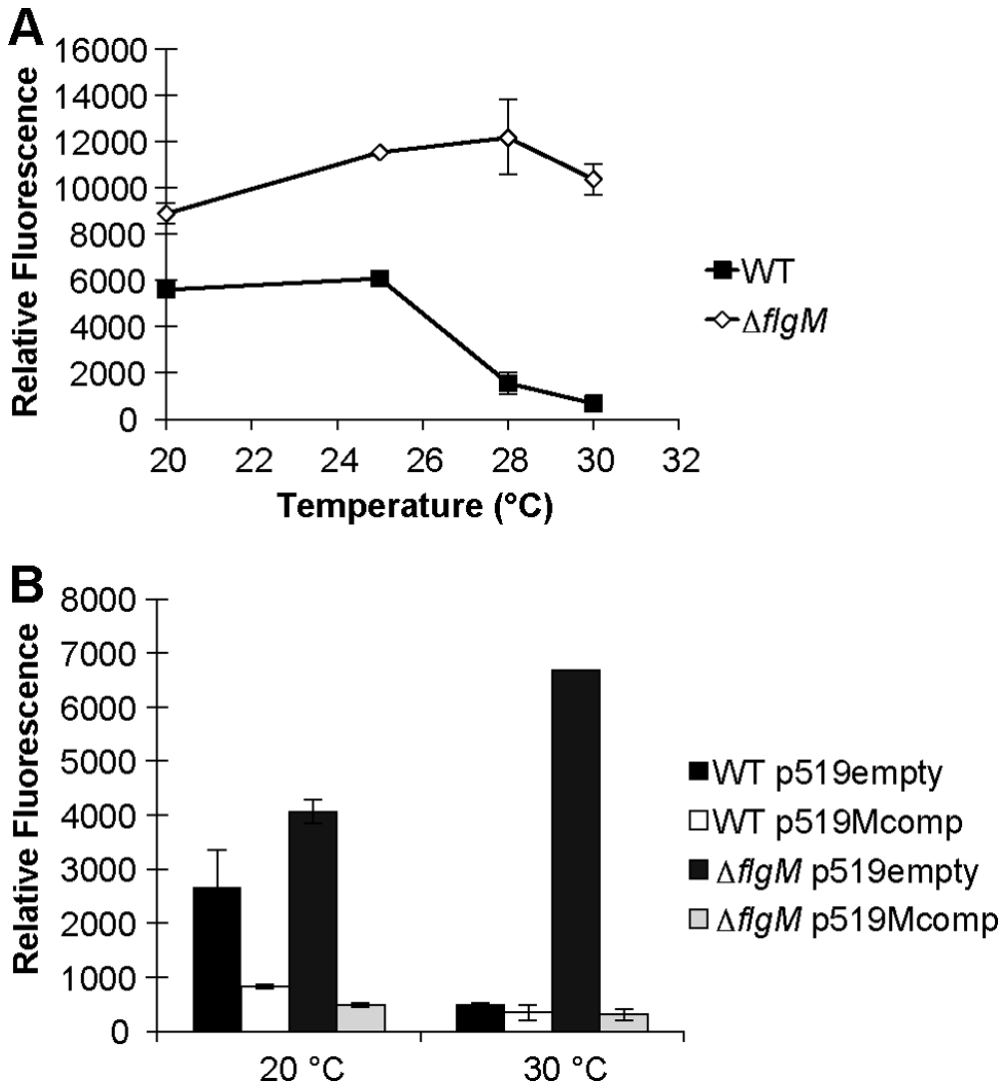
*salA* positively regulates *syfA*. Cell-normalized GFP fluorescence exhibited by wild type and various mutants of *Pseudomonas syringae* B728a harboring a *gfp* reporter gene fused either to *syfA* (A) or *syfR* (B) following 24 hours of incubation at 20°C. Vertical bars represent the standard deviation of the mean.

While we were unable to link *flgM* to the regulation of *syfA*, results from the transcriptome indicated that genes involved in the biosynthesis and transport of the phytotoxins syringomycin and syringopeptin, as well as their regulators, *salA*, *syrG*, and *syrF* were all more highly expressed at 20°C than at 30°C, similar to *fliC* and *syfA*. Because of the similarities in synthesis (lipopeptides synthesized by NRPSs) and temperature-dependent regulation between the former two phytotoxins and the latter biosurfactant, we posited that they shared a common regulator. We found that both *syfA* as well as *syfR* (a LuxR-type regulator required for syringafactin expression [20], [21]) expression was dependent on *salA* (Fig. 5). Additionally, as *salA* is positively regulated by *gacS* [25], *gacS* was also required for expression of *syfR* and *syfA*. Taken together, this data demonstrates that syringafactin expression is under *gac*-regulation via *salA*.

### Temperature Affects the Ability of *P. syringae* to Survive Desiccation Stress on Leaves

**Figure 6:**
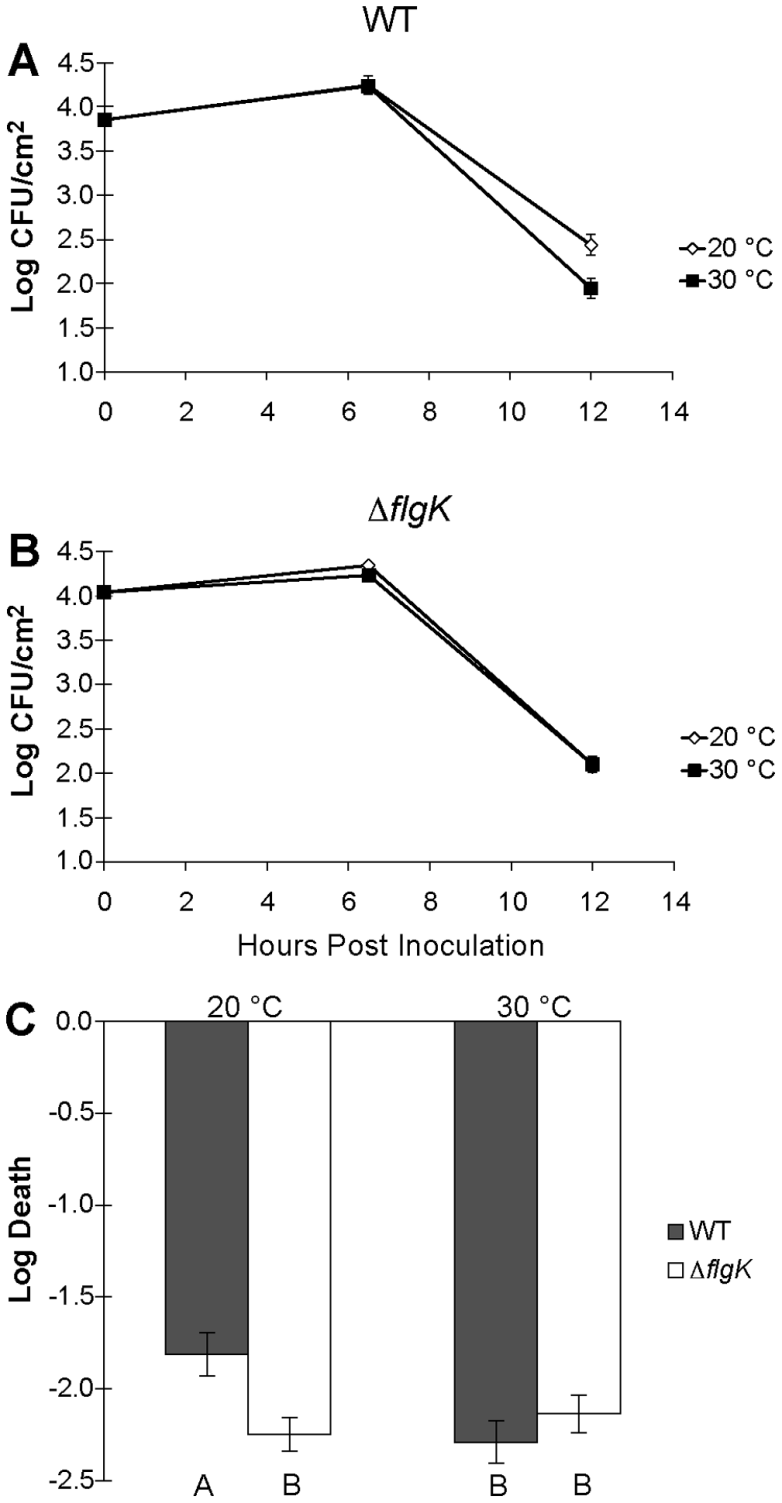
Temperature-dependent epiphytic survival. Epiphytic population size of wild type *Pseudomonas syringae* B728a (A) or a Δ*flgK* mutant (B) incubated at 20°C (open diamond) or 30°C (black square) for 6.5 hours at 100% RH prior to being exposed to desiccation at 26–28°C and 60–65% RH. Log-transformed population size decline was measured during the desiccation period that occurred between 6.5 and 12 hours after inoculation (C). This experiment was repeated several times with similar results. Treatments noted with the same letter do not differ significantly at a *p*-value ≤0.05 as determined by least significant difference test (LSD). The vertical bars represent the standard deviation of the mean. Error bars are present in panel B, but in some cases are small and obscured by the treatment symbol.

Given that motility is expected to be required for cells to access sites where environmental extremes such as desiccation stress could best be avoided, and that high temperatures appeared to strongly suppress the motility of *P. syringae*, we measured the temperature-dependent survival of *P. syringae* on leaves. We compared epiphytic populations of viable cells recovered from plants incubated under cool conditions (20°C, motility-inducing) and warm conditions (30°C, motility-suppressing) after inoculation. To minimize the confounding effect of temperature on the plant itself, as well as possible differences in population sizes of the bacterium on plants due to different growth rates at various temperatures, bacteria were only incubated on plants at the test temperature under moist conditions for 6 hours. After this brief period of incubation under near saturating humidity such that motility would be permitted, all plants were moved to a common area where leaves were allowed to dry with a constant temperature of 28°C. Bacterial cells incubated on cool, moist leaves survived the subsequent desiccating conditions an average of 3.2-fold better than cells that had been incubated on warm, moist leaves (Fig. 6). Importantly, a *flg*K mutant (which is defective in flagellar motility) survived poorly on both cool and warm leaves and exhibited similarly large declines in viable population sizes as observed for the WT strain incubated at 30°C. These results suggest that motility is required for stress survival on leaves and that the elevated motility of *P. syringae* on cool plants facilitated its acquisition of sites where desiccation survival was enhanced. In contrast, we found no evidence that syringafactin production contributed to enhanced survival on leaves incubated at cool temperatures under these conditions since the survival of a WT and a *syfA* mutant were similar on moist leaves incubated at a given temperature (data not shown). Together, these results suggest that bacterial cell behavior during the brief periods that leaves were moist modulated their ability to survive the subsequent desiccation stresses, and motility was a sufficient trait to account for this apparent stress avoidance.

### Global Transcriptome Analysis of *P. syringae* Incubated under Warm and Cool Conditions

As the effect of temperature on swimming and swarming motility was linked to a transcriptional response of genes encoding flagellin and syringafactin, we assessed the effect of temperature on the entire transcriptome of *P. syringae* by comparing the abundance of messages in cells incubated at 20°C and 30°C using Illumina next-generation sequencing of cDNAs. In total, 28.3% of the putative protein-encoding genes were differentially expressed between these two temperatures, with 338 genes more highly expressed at 20°C than at 30°C, while 1107 genes were more highly expressed at 30°C than at 20°C (Tables S1 and S2). Of the 338 genes that were up-regulated under cool conditions, 205 were induced more than 2-fold, while 133 were induced less than 2-fold. Of the 1107 genes that were up-regulated under warm conditions, 572 were induced more than 2-fold, while 535 were induced less than 2-fold. The differentially regulated genes were grouped into functional categories to determine if particular processes were more prominently temperature-dependent in *P. syringae*. Tables 2 and 3 list the functional categories in which genes preferentially expressed at 20°C and 30°C, respectively, were significantly over-represented. More functional categories were enriched at 20°C than at 30°C. The differential expression of genes in the following functional groups was particularly noteworthy:

**Table 2:** Functional gene categories preferentially up-regulated under cool growth conditions.

Category	*p*-value^a^
Polysaccharide synthesis and regulation	8.03E−05
Phage & IS elements	5.70E−14
Type VI secretion system	1.17E−13
Chemosensing & chemotaxis	4.18E−09
Translation	0.02
Flagellar synthesis and motility	9.32E−06
Phytotoxin synthesis andtransport	4.77E−10

Significance of functional category enrichment assessed using the hypergeometric distribution [77].

a Bonferroni corrected *p*-value.

**Table 3:** Functional gene categories preferentially up-regulated under warm growth conditions.

Category	*p*-value^a^
Hypothetical	0.04
Transcriptional regulation	8.73E−04
QAC metabolism and transport	1.56E−04
Chaperones/heat shock proteins	0.01

Significance of functional category enrichment assessed using the hypergeometric distribution [77].

a Bonferroni corrected *p*-value.

#### Polysaccharide synthesis and regulation

Genes in this functional category were predominantly induced at 20°C. Noteworthy were genes involved in alginate synthesis (*algE*, 1.9-fold; *algK*, 2.2-fold; *alg44* 2.4-fold; *alg8*, 2.1-fold; and *algD*, 3.0-fold), levan synthesis (*lscC*-1, 4.2-fold), and Psl polysaccharide synthesis (*pslA*, 3.7-fold; *pslB*, 3.5-fold; *pslD*, 2.1-fold; *pslE*, 2.8-fold; *pslF*, 3.4-fold; *pslG*, 2.6-fold; *pslH*-1, 2.3-fold; *pslI*, 3.0-fold; *pslJ*, 2.0-fold). Interestingly, there is a second copy of a gene putatively encoding levansucrase in the *P. syringae* genome (psyr_2103, *lscC*-2) that is induced by 2.1-fold at 30°C, exhibiting an opposite pattern of temperature-dependent expression compared to *lscC*-1. A few other genes in this functional category were also more highly expressed at 30°C, including 3 genes annotated as polysaccharide deacetylases with predicted functions as xylanases or chitin deacetylases (psyr_1809, 1.7-fold; psyr_2692, 2.2-fold; psyr_3937, 1.6-fold). *mucD,* encoding a negative regulator of AlgT (AlgU/σE) that controls alginate expression, also was more highly expressed at warm incubation temperatures (2.6-fold), which might explain the apparent induction of alginate at 20°C.

#### Phage and IS elements

Genes in this functional category were predominantly expressed at a higher level at 20°C. Prominent among such genes were those in two predicted prophage regions in the *P. syringae* genome corresponding to psyr_2761 to psyr_2832 (prophage region I) and psyr_4582 to psyr_4597 (prophage region II) identified with the Prophinder tool in ACLAME [26]. At least 22 genes in prophage region I were more highly expressed at 20°C than at 30°C, with a mean induction of 3.2-fold (range of 1.8 to 6.0-fold). However, 3 genes in prophage region I were more highly expressed at 30°C (psyr_2773, 2.0-fold; psyr_2791, 2.7-fold; psyr_2826, 1.7-fold). All 16 genes in prophage region II were more highly expressed at 20°C with a mean induction of 5.0-fold (range 1.7 to 7.3-fold).

#### Type VI secretion system

Most genes associated with the recently described type VI secretion system (T6SS) in *P. syringae* B728a [27], including genes encoding structural components of the secretion apparatus as well as ClpV, an ATPase putatively involved in driving export of secretion substrates, were more highly expressed at 20°C than at warmer temperatures. T6SS genes had a mean induction of 2.0-fold (range of 1.7- to 2.6-fold). However, 2 putative hcp homologs often associated with T6SS but not co-located with other T6SS genes, were both more highly expressed at 30°C (psyr_1935, 2.1-fold; psyr_4039, 2.9-fold).

#### Chemosensing and chemotaxis

Many genes in this functional category, including those encoding putative methyl-accepting chemotaxis proteins (15) as well as homologs of *cheV* (2), *cheW* (3), *cheB* (1), *cheA* (1), and an aerotaxis receptor, were more highly expressed at 20°C. The mean induction of these genes at 20°C was 3.3-fold (range of 1.7- to 11.9-fold). In contrast, 2 putative methyl-accepting chemotaxis proteins were more highly expressed at 30°C (psyr_0868 and psyr_1539, 2.0-fold and 1.8-fold, respectively).

#### Translation

Many genes encoding proteins associated with the 50S and 30S ribosomal subunits, as well as elongation factor Tu and G, were more highly expressed at 20°C than at 30°C (average of 2.0-fold induction with a range of 1.6- to 2.3-fold).

#### Transcriptional regulation

The genes encoding numerous DNA-binding transcriptional regulators belonging to the AraC, AsnC, DoeR, GntR, IclR, LacI, LuxR, LysR, MarR, TetR families, as well as putative response regulator partners of two component systems that also possess DNA binding domains were more highly expressed at 30°C (Table 4). Very few genes encoding putative transcriptional regulators were induced at 20°C, with the exception of 4 LuxR-type regulators that are involved in syringomycin, syringopeptin, and syringafactin regulation (*salA*, *syrG*, *syrF*, and *syfR*). Interestingly, those putative response regulator partner proteins that lack discernible DNA binding domains, were more likely to be induced at 20°C compared to 30°C (17.6% vs. 2.9%).

**Table 4:** Transcriptional regulators influenced by temperature.

Regulator Type	Annotated in the genome	Induced at 30°C(%)	Induced at 20°C(%)
LysR	74	21 (28.4)	1 (1.4)
GntR	27	12 (44.4)	0 (0.0)
TetR	27	9 (33.3)	0 (0.0)
LuxR	24	6 (25.0)	4 (16.7)
AsnC	7	2 (28.6)	0 (0.0)
AraC	19	7 (36.8)	0 (0.0)
IclR	5	1 (20.0)	0 (0.0)
DoeR	5	2 (40.0)	0 (0.0)
LacI	15	3 (20.0)	1 (6.7)
MarR	7	1 (14.3)	0 (0.0)
Response Regulator with putative DNA binding domain^a^	28	11 (39.3)	0 (0.0)
Response Regulatorwith out putative DNA binding domain	34	1 (2.9)	6 (17.6)

a This contains 10 Response regulator domains that are paired with LuxR DNA binding domains, 4 of which are induced at 30°C, none of which are induced at 20°C.

#### Quaternary ammonium compound (QAC) metabolism and transport

Genes encoding enzyme systems involved in choline, glycine betaine (GB), and carnitine import and metabolism were generally up-regulated at 30°C, while the *opuC* transporter, which is associated with osmoprotection [28], was modestly induced at 20°C (range of 1.6- to 1.8-fold). The *betT* transporter (associated with osmoprotection), along with *betX* and *caiX* solute binding proteins (associated with catabolism) which import betaine and carnitine, respectively, were induced by 2.3- to 2.8-fold at 30°C [29], [30]. All genes necessary for conversion of choline and glycine betaine to glycine were induced at 30°C, including *betAB* (range of 4.3 to 4.9-fold), *gbcAB* (range of 1.8 to 2.1-fold), *dgcAB* (range of 2.0- to 2.3-fold), and *soxBDAG* (range of 1.7- to 2.3-fold) [31]. *dhcA* and *dhcB*, both predicted to encode proteins that catalyze the second catabolic step in carnitine breakdown, were more highly expressed at 20°C (range of 1.9- to 2.1-fold), while their regulator (*dhcR*) was expressed 2.8-fold higher at 30°C than at 20°C. As DhcR induces transcription of *dhcAB* in the presence of 3-dehydrocarnitine (an intermediate in the catabolism of carnitine), these results might suggest that elevated growth temperature increases carnitine catabolism, but that the inducing intermediate was not present in this experiment.

#### Hypothetical

Surprisingly, the proportion of genes with no known function that were more highly expressed at high temperatures than at cooler temperatures was much higher than their proportion in the genome. Such genes included both those encoding proteins that possess domains of unknown function, as well as those without such conserved domains.

In addition to the general categories noted above, genes involved in auxin biosynthesis were temperature regulated. Both homologs of *iaaM* (psyr_1536, psyr_4667) and *iaaH* (psyr_2208, psyr_4268), involved in biosynthesis of indole-acetic acid (IAA) were induced at 30°C, as were genes involved in the biosynthesis of tryptophan, a precursor for IAA.

## Discussion

The motility of *P. syringae*, like some other bacteria, is very strongly influenced by temperature. Both swimming and swarming motility are inhibited by incubation at 30°C and this is apparently due to the suppression of expression of flagellar components required for both swimming and swarming motility, as well as reduced expression of syringafactin that contributes to swarming. In addition to syringafactin, *P. syringae* B728a produces a second biosurfactant, 3-(3-hydroxyalkanoyloxy) alkanoic acid (HAA), which is synthesized by RhlA. In contrast to *syfA*, *rhlA* (psyr_3129) was not thermo-regulated at the transcript level (Table S1), potentially indicating that regulation of this surfactant is independent of temperature. Temperature-regulated swarming motility and surfactant production has also been observed in *Pseudomonas putida* KT2440, a non plant-pathogenic, soil-associated Pseudomonad, which moves at 22°C but not 30°C [32]. Interestingly, biosurfactant production has not been reported for this strain and its swarming motility is not flagellar-dependent, but rather type IV pilus-dependent [32]. In contrast, another strain, *P. putida* PCL1445, produces the biosurfactants putisolvin I and II, required for swarming, in a temperature-dependent manner [33], [34], [35], but neither temperature-regulated flagellar expression nor swimming motility has been reported. The production of these biosurfactants is, however, dependent on the *gacS*/*gacA* two component signaling system, both of which are more highly expressed at 11°C than at 28°C or 32°C [34], [35]. In contrast to this strain, the expression of neither *gacS* nor *gacA* in *P. syringae* B728a was influenced by temperature under the conditions tested, as determined by the transcriptome results.

Inhibition of flagellar motility occurs at the optimal growth temperature of *P. syringae* as in many bacteria. This phenomenon has been studied most extensively in animal pathogens [36] but it seems likely that the biological relevance for such a phenotype differs in bacteria colonizing such different habitats. *flgM*, encoding an anti-sigma factor known to inhibit the activity of FliA in numerous organisms, also was required for thermo-repression of *fliC* in *P. syringae*. The patterns of thermo-regulation in *P. syringae* seem similar to that of flagellar thermo-regulation in *Yersinia enterocolitica*, whose motility is regulated in a manner that generally conforms to the three-tiered regulatory hierarchy established in *E. coli* and *S. typhimurium* [37]. In *Y. enterocolitica* flagellar-mediated motility is expressed at 25°C but repressed at 37°C, while plasmid-encoded virulence factors are oppositely regulated [38]. Thermo-regulation occurs at the level of *fliA* expression, while expression of other class I and class II flagellar genes are insensitive to incubation temperature [37], [39], [40]. Disruption of *fliA* leads to reduced or abolished thermo-regulation of plasmid-encoded virulence factors, and therefore is thought to be a key mediator of the temperature response [37]. In *P. syringae*, however, we found that *fliA* transcription itself was insensitive to incubation temperature, suggesting instead that FlgM mediates thermo-regulation. Indeed, the FlgM-FliA interaction in *Campylobacter jejuni* is temperature dependent, being destabilized at 42°C compared to 32°C or 37°C [41]. *Campylobacter* is unique in that it is one of the few genera where motility and flagellin expression is maximal at its growth temperature optimum (42°C) [41], [42]. It is not clear whether the FliA-FlgM interaction in *P. syringae* is itself temperature sensitive or if other factors mediate a temperature-sensitive interaction of these two components. If this interaction is itself temperature sensitive, our results would suggest that the interaction in *P. syringae* is stabilized with increasing temperature, opposite to that in *Campylobacter*. It is unclear if thermo-regulation of FliA activity in *P. syringae* contributes to thermo-regulation of non-flagellar genes similar to its role in *Y. enterocolitica*. However, the fact that *flgM* is not required for thermo-repression of *syfA*, coupled with the observation that the expression of over 1000 genes is significantly influenced by incubation temperature indicates that at least one, if not several, other genes contribute to thermo-regulation in *P. syringae*.

Syringomycin production was previously shown to be thermo-regulated [43], which is consistent with our results indicating that the genes involved in the biosynthesis, transport and regulation of syringomycin and syringopeptin were all thermo-regulated in the transcriptome. Both *syfR* and *syfA* are positively regulated by *salA* (Fig. 6), which is also a positive regulator of syringomycin and syringopeptin [44], [45]. While it is not clear at this point which gene(s) are required for thermo-regulation of these three lipopeptides, it seems likely that they may share a common thermo-regulator. Additionally, it appears that thermo-regulation of the flagellum and thermo-regulation of syringafactin are independent of each other because mutations affecting thermo-regulation of *syfA* do not influence thermo-regulation of *fliC* (Hockett and Lindow, unpublished).

The apparent temperature-dependent expression of a variety of EPS species in *P. syringae* suggests that such molecules play a very context-dependent role in the fitness of this bacterium. In addition to alginate production, genes involved in producing other EPS species such as Psl and levan were, in general, strongly suppressed at 30°C. In *P. syringae* pv. glycinea, levan production is also thermo-regulated, accumulating at 18°C but not 28°C [46]. Similar to our observation that *lscC*-2 was oppositely regulated compared to the majority of genes involved in EPS synthesis, being more highly expressed at 30°C than 20°C, LscC in *P. syringae* pv. glycinea is more abundant within the cell at 28°C compared to 18°C [46]. The enzyme is not, however, exported at the elevated temperature, which restricts levan formation to cool temperatures. Levansucrase enzyme location may be similarly regulated in *P. syringae* B728a. Psl, a recently discovered EPS involved in biofilm formation in *Pseudomonas aeruginosa* [47], [48], is hypothesized play a similar role in *P. syringae* [49], [50]. There may be a conflict in the roles of Psl and motility in biofilm formation as flagellar-mediated motility apparently contributes to initial attachment of cells to surfaces [51], while the expression of EPS could interfere with motility of *P. syringae.* Further investigation will be required to directly address conflicts arising from increased expression of genes mediating motility along with genes mediating EPS production. We presume that during our *in planta* assays, cells that became motile under the cool conditions accessed preferred sites of colonization (base of glandular trichomes, intercellular grooves between epidermal cells) that facilitated their subsequent desiccation tolerance or enabled them to escape such stresses. A non-mutually exclusive possibility is that such cool conditions stimulated biofilm formation due to both enhanced motility and EPS production and that survival was enhanced on the leaf surface itself [52], [53]. Further directed studies will be required to discern whether motility itself or its role in biofilm formation (or both) contribute to cool temperature induced desiccation tolerance.

It was noteworthy that nearly all of the known uptake systems for QAC compounds, whether for their accumulation for osmoprotection or for their catabolism were more highly expressed at 30°C. These results suggest that increased import capacity of these compounds would allow the cell to rapidly accumulate compatible solutes by repressing expression of the catabolic enzymes, should water limitation occur (conditions which did not occur in our experiment). Alternatively, *P. syringae* may anticipate the increased abundance of QACs in the plant environment using temperature as a cue as tomato plants were found to accumulate QACs at temperatures above 25°C [54]. Indeed, other researchers have observed that *P. syringae* does not maintain a measurable glycine betaine pool at 30°C, but does maintain such a pool at 22–25°C, which would be consistent with increased catabolism (M. Wargo, personal communication and [55]).

One of the most obvious effects of temperature on *P. syringae* that is linked to its behavior on plants is the thermo-regulation of motility. The lower survival of cells of *P. syringae* exposed to warm temperatures during moist incubation prior to desiccation stress is consistent with previous work that showed that non-motile mutants of this species were less resistant to desiccation and UV stress *in planta* [6]. While there have been numerous descriptions of thermo-regulated motility in animal pathogens, there have been only two studies to date that have linked temperature with the motility of plant pathogenic bacteria [7], [56]. Flagellar motility of *Erwinia amylovora,* which is suppressed at warm temperatures, appeared to play little role in the infection and disease process, as its virulence was independent of temperature. In addition to thermo-regulation of motility, *E. amylovora* expresses amylovoran, an EPS required for virulence, and type III secretion system-associated genes in a temperature dependent manner, with greater expression at cooler temperatures [57], [58], [59], [60]. In contrast to *E. amylovora*, symptom development in lilac inoculated with *P. syringae* isolates was lower on plants incubated at temperatures above 24°C than at cooler temperatures [61]. However, due to the invasive method of inoculation used in this study, motility may not have been required for infection and the effect of temperature on the motility of the pathogen was not assessed. While plant disease symptoms caused by other *P. syringae* strains also have been noted to be higher under cool conditions, such effects on virulence cannot be linked to the motility of the pathogen. For example, pre-incubation of *P. syringae* pv. glycinea PG4180 at 18°C enhanced subsequent *in planta* multiplication and disease symptoms compared to when preincubated at 28°C [62]. This temperature-dependent interaction was dependent on production of the toxin coronatine, which is produced only at cool temperatures. It has been hypothesized that the increased occurrence and severity of bacterial plant disease often seen under cool, wet weather in temperate climates is a result of increased opportunity for invasion due to increased water availability that coincides with cool temperatures [18]. Our results, in conjunction with previous results, suggest that temperate pathogens are also more invasive under these conditions because they more highly express motility-related traits under cool conditions.

This work supports the notion of *P. syringae* as a conservative pathogen that coordinates motility with environmental conditions in a way that will be optimally productive for the pathogen. It also suggests that the pathogen exhibits anticipatory behaviors, using high temperature as an indicator that water availability may soon be limited, and thus dictating that it express such a trait in a conservative manner. This strategy would maximize its fitness over the long run by avoiding risks associated with short-term exposure to stresses such as desiccation that could be avoided.

## Materials and Methods

### Bacterial Strains, Plasmids, Culture Media, and Growth Conditions

*Pseudomonas syringae* pv. syringae B728a [63] was routinely cultured in King’s medium B (KB) broth, or on KB plates supplemented with 1.5% (w/v) Difco agar technical (BD, Sparks, MD) at 28°C [64]. Escherichia strains TOP10 (Life technologies, Carlsbad, CA) and S17-1 [65] were cultured in Luria-Bertani (LB) medium broth, or on LB plates supplemented with 1.5% (w/v) Difco agar technical at 37°C. Antibiotics were used at the following final concentrations: rifampicin, 100 µg/mL; kanamyacin, 50 µg/mL; gentamicin, 15 µg/mL; spectinomycin 20 µg/mL; tetracycline, 15 µg/mL; nitrofurantoin (NFT), 30 µg/mL. Natamycin (antifungal) was used at 20 µg/mL. Strains and plasmids used in this work are listed in Table S3. Primers used in this work are listed in Table S4. The temperature of agar plates were routinely monitored using a CZ-IR thermometer (ThermoWorks, Lindon, UT). Incubator temperatures and relative humidity were routinely monitored using HOBO data loggers (Onset, Bourne, MA).

### Detection of Biosurfactants

Biosurfactants were detected with an atomized oil assay similar to previously described [20]. Bacterial cultures were grown overnight and diluted into fresh medium the following morning and grown for 2–4 hours. Cultures were then washed once with 10 mM KPO_4_ and resuspended to a concentration of 2.0×10^8^ CFU/mL, as determined by turbidity (OD_600_). 5 µL of resuspended culture was spotted onto KB plates and incubated for 20–30 hours at either 20°C or 30°C, then sprayed with a mist of mineral oil. The diameter of the visible halo of brighter oil drops was measured and the area of the producing bacterial colony was calculated and subtracted from that of the surfactant halo to yield the adjusted halo area.

### Swimming and Swarming Assays

Swimming media (50% KB containing 0.25% agar) was allowed to dry overnight on the bench top (unstacked). Bacterial cultures were incubated overnight (28°C with shaking at 200 RPM) in KB broth amended with the proper antibiotics. Cultures were then diluted into fresh media and allowed to incubate for 2–4 hours and cells recovered by centrifugation and washed once with 10 mM KPO_4_ buffer before being resuspended to a final concentration of 2.0×10^8^ CFU/mL and inoculated by stabbing with a sterile toothpick. Plates were incubated for 20–30 hours prior to observation. Swarming assays were performed similar to swimming assays except 5 ul of bacterial suspensions were spot inoculated singly onto the center of swarming plates (undiluted KB containing 0.4% agar).

### Transcriptional Reporter Assays

Transcriptional reporter assays were performed similar to other studies [66]. Briefly, culture spots were resuspended in 10 mM KPO_4_ buffer and diluted to a final OD_600_ of 0.1–0.2. GFP fluorescence intensity was determined using a TD-700 fluorometer (Turner Designs, Sunnyvale, CA) with a 486-nm-band-pass excitation filter and a 510- to 700-nm combination emission filter. Relative fluorescence was determined by normalizing the arbitrary fluorescence units to optical density.

### RNA Isolation and qRT-PCR

Bacterial cultures were harvested in 1.0 mL of RNA *later* (Life Technologies; Carlsbad, CA) and stored at 4°C for no longer than one week prior to RNA isolation. Total RNA was isolated using TRIzol® reagent (Life Technologies) similar to manufacturer’s protocol except differing in the following ways: homogenized cells were incubated at 65°C for 10 minutes in TRIzol® prior to addition of chloroform; after adding chloroform, samples were incubated at RT for 15 minutes; RNA was precipitated in isopropanol at −80°C for 20 minutes. Washed RNA was resuspended in 30–50 µl of RNAsecure™ (Life Technologies), according to manufacturer’s protocol. To remove contaminating genomic DNA, RNA was treated with TURBO DNA-*free*™ (Life Technologies) according to manufacturer’s protocol. DNase-treated samples were either cleaned using the DNase inactivating reagent included in the TURBO DNA-*free*™ kit, or were column purified using RNeasy Mini Kit (QIAGEN; Valencia, CA). The absence of contaminating DNA was confirmed by performing a PCR reaction using Phusion® High-Fidelity DNA Polymerase (New England Biolabs; Ipswich, MA) with either DNase treated or untreated samples using *rpoD-*RT-S and AS primers (Table S4). RNA purity and abundance was routinely assessed using an ND-1000 spectrophotometer (Thermo Scientific; Lafayette, CO). cDNA was generated from 0.5 or 10 µg of DNase-treated RNA, using SuperScript® II reverse transcriptase (Life Technologies) with random primers (Life Technologies). qPCR was performed using a 7300-Real-Time PCR System (Life Technologies) with QuantiTect SYBR Green I (QIAGEN) on 5 or 10 µl of 1∶1000 diluted cDNA. Samples not treated with reverse transcriptase were routinely included, which showed no significant increase in fluorescence following 35 cycles. Amplification efficiencies were determined using LinReg [67]. Amplification efficiencies were determined to be 95% of maximum, and were consistent between gene targets. Relative expression was calculated according to the ΔΔCt method with a base of 1.9 to account for amplification efficiency. *rpoD* and psyr_3981, encoding pseudouridine synthase which was found to be stably expressed under numerous conditions and in numerous mutant backgrounds (R. Scott and K. Hockett, unpublished data), were used as endogenous controls. qRT-PCR data normalized to either endogenous control gave similar results; results normalized to *rpoD* are shown.

### mRNA Sequencing

Total RNA was harvested as described above. Following total RNA isolation, 16S and 23S rRNA was removed using Ribo-Zero™ rRNA removal Kit (Gram-Negative Bacteria) (Epicentre, Madison, WI) according to the manufacturer’s protocol. Enriched mRNA samples were assayed with a 2100 Bioanalyzer (Agilent; Santa Clara, CA) to confirm removal of rRNA. Quantification of mRNA and dsDNA was routinely performed with qBit RNA and dsDNA HS assays (Life Technologies), respectively, according to the manufacturer’s protocol. Ambion fragmentation reagent (Life Technologies) was used to generate 100–200 nucleotide fragments from enriched mRNA. Fragmented RNA was isolated by ethanol precipitation with glycogen amendment, followed by resuspension in RNase free water. First strand cDNA synthesis was performed using random primers (Life Technologies) and Superscript II reverse transcriptase (Life Technologies), followed by second strand synthesis using RNase H and DNA pol I (Life Technologies). dsDNA was routinely purified using AMPure XP beads (Beckman Coulter, Brae, CA) with PEG 6000 added to a final concentration of 6.5% (w/v). cDNA end repair was performed using a combination of Klenow DNA polymerase, T4 DNA polymerase, and T4 polynucleotide kinase (New England Biolabs, Ipswich, MA). An A tail was added to end-repaired fragments using Klenow exo minus with 250 µM ATP (final concentration) (New England Biolabs). Illumina adapters were ligated to A-tailed dsDNA fragments using T4 DNA ligase (New England Biolabs), with PEG 6000 addition to 5.0% (w/v) final concentration. Adapter-ligated fragments were amplified using Illumina barcoded primers with Phusion DNA polymerase (New England Biolabs). Amplified fragments were purified using AMPure XP beads without PEG addition. Fragment sizes were confirmed to be in the size range of 200–300 nucleotides using the 2100 Bioanalyzer. Sequencing was performed using the Illumina HiSeq 2000 for 50 base pair reads through the Vincent J. Coates Genomics Sequencing Laboratory. Three biological repeats were sequenced per temperature treatment on three separate flow cells. Reads were aligned to the *P. syringae* pv. syringae B728a genome (downloaded from the Integrated Microbial Genomes website) using bwa [68] allowing for a maximum of three mismatches between a given read and the reference genome. The number of reads that overlapped with a given gene was counted using a custom script. Differential expression of genes and statistical significance were assessed using edgeR [69]. Briefly, significance was established by comparing gene expression levels, normalized by the trimmed mean of M-values (TMM) [70], between three biological replicates incubated at either 20°C or 30°C (six samples total) using an empirical Bayes estimation and exact tests based on the negative binomial distribution. Genes were considered significantly differentially regulated if the p-value (after adjustment for multiple comparisons) for a difference in expression between the two treatments was less than or equal to 0.05. Expression data is available at the Integrated Microbial Genome website (http://img.jgi.doe.gov).

### Deletion of *flgK*

A deletion mutant of *flgK* was constructed by cloning approximately 1 kb fragments upstream and downstream of *flgK* into pENTR/D-TOPO:MCS-Kan [71]. The region downstream of *flgK* was amplified using the primers flgKe-xhoF and flgKe-xbaR, digested with *XhoI* and *XbaI* and ligated into pENTR/D-TOPO:MCS-Kan. The region upstream of *flgK* was amplified using the primers flgKs-hindF and flgKs-speR, digested with *HindIII* and *SpeI*, and ligated into pENTR/D-TOPO:MCS-Kan. The resulting region containing both flanking sequences and *npt2* driving kanamycin resistance were transferred to pLVC/D [72] via a clonase LR reaction (Invitrogen). The resulting plasmid was isolated and electroporated into *E. coli* S17-1 for conjugal transfer. Both *E. coli* and *P. syringae* were grown individually overnight on plates, then mated overnight. Initial transformants were isolated on KB plates containing rifampin, kanamycin and tetracycline. Deletion mutants were selected that were kanamycin resistant but tetracycline sensitive. Deletions were confirmed by PCR amplification, which verified that the kanamycin cassette had replaced *flgK*.

### Deletion and Complementation of *flgM*

A markerless *flgM* deletion strain was constructed using an overlap extension PCR protocol similar to that of other studies [73]. Regions of approximately one kilobase of sequence upstream and downstream of *flgM* were amplified using 5′-S-*flgM*, 5′-AS-*flgM*, 3′-S-*flgM*, and 3′-AS-*flgM*. These fragments were combined with a kanamycin resistance cassette flanked by FLP recombinase target sites (kan-FRT) amplified from pKD13 [74] using primers pKD-13-site-4-AS and pKD4/13-site-1-S. The upstream, downstream, and kan-FRT fragments were combined into a single PCR reaction which was cycled for 15 amplification cycles in the absence of primers, followed by 20 amplification cycles with 5′-S-*flgM* and 3′-AS-*flgM* primers added. The resulting fragment was cloned into the suicide vector pTOK2T [29], creating p*flgM*-KO, which was transformed into the *E. coli* mating strain, S17-1. S17-1 harboring p*flgM*-KO was mated with *P. syringae* B728a overnight on KB, followed by transfer onto KB amended with kanamycin and NFT (counter selection for *E. coli*). Single colonies were streaked onto KB amended with either rifampicin and kanamycin or rifampacin and tetracyline. Colonies displaying kanamycin resistance and tetracycline sensitivity were screened for replacement of *flgM* with the kanamycin resistance cassette using PCR with the external and internal primer sets, 5′-S-*flgM* +3′-AS-*flgM* and *flgM*-coding-S+*flgM*-coding-AS, respectively. A spectinomycin-resistant derivative of pFLP2 [66], [75] was electroporated into the kanamycin resistant, Δ*flgM.* pFLP2-containing colonies were screened for kanamycin sensitivity. A single kanamycin sensitive isolate was grown in KB broth over night in the absence of selection, yielding a markerless Δ*flgM* strain.

To construct p519Mcomp, p519nGFP was digested with XbaI and EcoRI, followed by treatment with T4 DNA polymerase (to create blunt ends). The digested/blunted vector was ligated to a PCR fragment containing *flgM* with its promoter region, amplified with *flgM*-5′-S-complement and *flgM*-3′-AS-complement. p519empty was constructed by self-ligating the digested/blunted vector.

### Plant Assays

Three week old bean plants (*Phaseolus vulgaris* cv. Blue Lake 274) were acclimated to 30°C for 48 hours on a long day (16 hours light/8 hours dark) light regime prior to inoculation. Bacterial strains were grown over night at 30°C on KB to suppress motility. Cultures were resuspended in 10 mM KPO_4_ and diluted to a final concentration of 10^6^ CFU/mL in sterile, distilled water. Cultures were spray inoculated onto plants until dripping, and bagged to maintain high relative humidity. Inoculated plants were incubated at either 20°C or 30°C for 6 hours, prior to being unbagged and moved to a common incubation area (28°C, 50–60% relative humidity) for an additional six hours of incubation. At the time of transfer, condensation was present on the interior surfaces of plant bags, indicating near saturating humidity under both temperature treatments. For each treatment three primary leaves were sampled immediately after inoculation, and ten primary leaves were sampled immediately after the plants were un-bagged and again six hours later. Harvested leaves were sonicated in washing buffer (100 mM KPO_4_ buffer, 0.1% peptone) for 5 minutes, followed by dilution plating onto KB plates amended with rifampicin and natamycin. Bacterial population size was normalized for leaf surface area determined by analysis of digital images of leaves using ImageJ software [76]. The log death value was calculated as the difference between the log-transformed, leaf-area normalized population size determined immediately after the plants were un-bagged and those at the conclusion of the experiment.

## Supporting Information

Figure S1 **Temperature-dependent biosurfactant production.** Area of biosurfactant-coverage on agar plates produced by either wild type *Pseudomonas syringae* B728a (grey bars) or a Δ*flgM* mutant (white bars) when grown at either 20°C or 30°C. The vertical bars represent the standard deviation of the mean.
(TIF)

Table S1 **Thermo-regulated genes in** ***P. syringae.***
(CSV)

Table S2 **Functional categories of thermo-regulated genes.**
(DOCX)

Table S3 **Bacterial strains and plasmids.**
(DOCX)

Table S4 **Primers used in cloning and qRT-PCR.**
(DOCX)
