# Supplementary figures and images for: Thermo-Regulation of Genes Mediating Motility and Plant Interactions in Pseudomonas syringae
Source: PLoS One. 2013 Mar 19;8(3):e59850. doi: 10.1371/journal.pone.0059850 (PMC3602303; doi:10.1371/journal.pone.0059850)

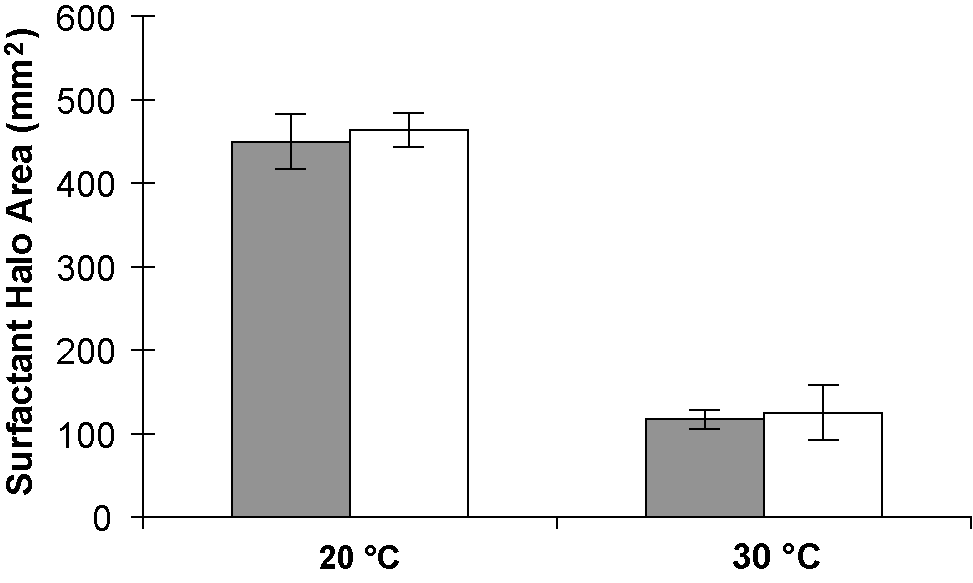

Supplement: Figure S1 — Temperature-dependent biosurfactant production. Area of biosurfactant-coverage on agar plates produced by either wild type Pseudomonas syringae B728a (grey bars) or a ΔflgM mutant (white bars) when grown at either 20°C or 30°C. The vertical bars represent the standard deviation of the mean. (TIF) [file pone.0059850.s001.tif]
