# Supplementary material for: Thermo-Regulation of Genes Mediating Motility and Plant Interactions in Pseudomonas syringae
Source: PLoS One. 2013 Mar 19;8(3):e59850. doi: 10.1371/journal.pone.0059850 (PMC3602303; doi:10.1371/journal.pone.0059850)
Supplement: Table S2 — Functional categories of thermo-regulated genes. (DOCX) [file pone.0059850.s003.docx]

**Table S2:**  Functional categories of thermo-regulated genes

| **Functional Category** | **# of Genes^a^** | **% of Total genes** | **% of Genes Temperature Sensitive^b^** | **Cold Induced** | **% of Cold Induced Genes** | **Hot Induced** | **% of Hot Induced Genes** |
| --- | --- | --- | --- | --- | --- | --- | --- |
| Type VI secretion system | 28 | 0.55 | 67.9 | 18 | 5.3 | 1 | 0.1 |
| QAC metabolism and transport | 42 | 0.82 | 66.7 | 5 | 1.5 | 23 | 2.1 |
| Toxin-Antitoxin system | 6 | 0.12 | 66.7 | 0 | 0.0 | 4 | 0.4 |
| Phytotoxin synthesis and transport | 23 | 0.45 | 60.9 | 14 | 4.1 | 0 | 0.0 |
| Cold shock proteins | 5 | 0.10 | 60.0 | 1 | 0.3 | 2 | 0.2 |
| Chaperones/Heat shock proteins | 28 | 0.55 | 57.1 | 1 | 0.3 | 15 | 1.4 |
| TAT secretion system | 10 | 0.20 | 50.0 | 0 | 0.0 | 5 | 0.5 |
| Plant-associated proteins | 11 | 0.22 | 45.5 | 0 | 0.0 | 5 | 0.5 |
| Amino acid metabolism and transport (GABA) | 7 | 0.14 | 42.9 | 0 | 0.0 | 3 | 0.3 |
| Phage & IS elements | 125 | 2.45 | 42.4 | 37 | 10.9 | 16 | 1.4 |
| Special | 12 | 0.24 | 41.7 | 0 | 0.0 | 5 | 0.5 |
| Transport (peptides) | 36 | 0.71 | 38.9 | 3 | 0.9 | 11 | 1.0 |
| Siderophore synthesis and transport | 76 | 1.49 | 38.2 | 10 | 3.0 | 19 | 1.7 |
| Polysaccharide synthesis and regulation | 49 | 0.96 | 36.7 | 11 | 3.3 | 7 | 0.6 |
| Type III secretion system | 49 | 0.96 | 36.7 | 0 | 0.0 | 18 | 1.6 |
| Carbohydrate metabolism and transport | 117 | 2.29 | 35.0 | 8 | 2.4 | 33 | 3.0 |
| Transcriptional regulation | 200 | 3.92 | 34.5 | 0 | 0.0 | 69 | 6.2 |
| Chemosensing & chemotaxis | 73 | 1.43 | 34.2 | 23 | 6.8 | 2 | 0.2 |
| Oxidative stress tolerance (Antioxidant enzyme) | 15 | 0.29 | 33.3 | 2 | 0.6 | 3 | 0.3 |
| Flagellar synthesis and motility | 48 | 0.94 | 33.3 | 15 | 4.4 | 1 | 0.1 |
| Outer membrane proteins | 24 | 0.47 | 33.3 | 4 | 1.2 | 4 | 0.4 |
| Fatty acid metabolism | 55 | 1.08 | 30.9 | 1 | 0.3 | 16 | 1.4 |
| Hypothetical^c^ | 1219 | 23.87 | 29.9 | 57 | 16.9 | 307 | 27.7 |
| Transport | 101 | 1.98 | 29.7 | 3 | 0.9 | 27 | 2.4 |
| Mechanosensitive ion channel | 7 | 0.14 | 28.6 | 0 | 0.0 | 2 | 0.2 |
| Secretion/Efflux/Export | 100 | 1.96 | 28.0 | 6 | 1.8 | 22 | 2.0 |
| Sulfur metabolism and transport | 61 | 1.19 | 27.9 | 0 | 0.0 | 17 | 1.5 |
| Secondary metabolism | 26 | 0.51 | 26.9 | 3 | 0.9 | 4 | 0.4 |
| Organic acid metabolism and transport | 105 | 2.06 | 26.7 | 4 | 1.2 | 24 | 2.2 |
| Cyclic di-GMP cyclase proteins | 34 | 0.67 | 26.5 | 2 | 0.6 | 7 | 0.6 |
| Compatible solute synthesis | 19 | 0.37 | 26.3 | 0 | 0.0 | 5 | 0.5 |
| Unannotated^d^ | 1007 | 19.72 | 26.2 | 36 | 10.7 | 226 | 20.4 |
| Pili synthesis and regulation | 51 | 1.00 | 25.5 | 2 | 0.6 | 11 | 1.0 |
| Transcription - Sigma factor | 16 | 0.31 | 25.0 | 1 | 0.3 | 3 | 0.3 |
| Phosphate metabolism and transport | 20 | 0.39 | 25.0 | 2 | 0.6 | 3 | 0.3 |
| Amino acid metabolism and transport | 238 | 4.66 | 24.4 | 18 | 5.3 | 40 | 3.6 |
| Energy generation | 83 | 1.63 | 24.1 | 7 | 2.1 | 13 | 1.2 |
| LPS synthesis and transport | 38 | 0.74 | 23.7 | 1 | 0.3 | 8 | 0.7 |
| Nitrogen metabolism | 57 | 1.12 | 22.8 | 2 | 0.6 | 11 | 1.0 |
| Stress resistance | 41 | 0.80 | 22.0 | 0 | 0.0 | 9 | 0.8 |
| Polyamine metabolism and transport | 19 | 0.37 | 21.1 | 0 | 0.0 | 4 | 0.4 |
| Transport (inorganic ions) | 48 | 0.94 | 20.8 | 1 | 0.3 | 9 | 0.8 |
| Osmosensing & regulation | 5 | 0.10 | 20.0 | 1 | 0.3 | 0 | 0.0 |
| Degradation of xenobiotics | 15 | 0.29 | 20.0 | 0 | 0.0 | 3 | 0.3 |
| Transcription | 10 | 0.20 | 20.0 | 2 | 0.6 | 0 | 0.0 |
| RNA degradation | 16 | 0.31 | 18.8 | 3 | 0.9 | 0 | 0.0 |
| Cofactor metabolism | 152 | 2.98 | 18.4 | 1 | 0.3 | 27 | 2.4 |
| Transport (organic compounds) | 11 | 0.22 | 18.2 | 0 | 0.0 | 2 | 0.2 |
| Light and oxygen sensing | 11 | 0.22 | 18.2 | 0 | 0.0 | 2 | 0.2 |
| Proteases | 17 | 0.33 | 17.6 | 1 | 0.3 | 2 | 0.2 |
| Signal transduction mechanisms | 40 | 0.78 | 17.5 | 4 | 1.2 | 3 | 0.3 |
| Translation | 132 | 2.59 | 16.7 | 19 | 5.6 | 3 | 0.3 |
| Post-translational modification | 12 | 0.24 | 16.7 | 0 | 0.0 | 2 | 0.2 |
| Glutathione metabolism | 18 | 0.35 | 16.7 | 1 | 0.3 | 2 | 0.2 |
| Quorum regulation | 6 | 0.12 | 16.7 | 0 | 0.0 | 1 | 0.1 |
| Iron metabolism and transport | 27 | 0.53 | 14.8 | 0 | 0.0 | 4 | 0.4 |
| Phospholipid metabolism | 36 | 0.71 | 13.9 | 4 | 1.2 | 21 | 1.9 |
| Nucleotide metabolism and transport | 83 | 1.63 | 12.0 | 3 | 0.9 | 7 | 0.6 |
| Replication and DNA repair | 97 | 1.90 | 11.3 | 0 | 0.0 | 11 | 1.0 |
| Oxidative stress tolerance | 11 | 0.22 | 9.1 | 1 | 0.3 | 0 | 0.0 |
| Terpenoid backbone synthesis | 12 | 0.24 | 8.3 | 0 | 0.0 | 1 | 0.1 |
| Peptidoglycan/cell wall polymers | 29 | 0.57 | 6.9 | 0 | 0.0 | 2 | 0.2 |
| Iron-sulfur proteins | 14 | 0.27 | 0.0 | 0 | 0.0 | 0 | 0.0 |
| Oxidative stress tolerance (antioxidant enzyme) | 2 | 0.04 | 0.0 | 0 | 0.0 | 0 | 0.0 |
| Cell division | 21 | 0.41 | 0.0 | 0 | 0.0 | 0 | 0.0 |
| Total | 5106 | 100.0 | 28.3 | 338 | 100.0 | 1107 | 100.0 |

^a^Total number of genes within functional category

^b^Percentage of functional category that is temperature sensitive

^c^Hypothetical refers to predicted proteins with no discernable domain or motifs

^d^Unannotated refers to predicted proteins with discernable domains or motifs, but lack sufficient evidence to place within a functional category
