# Supplementary material for: Thermo-Regulation of Genes Mediating Motility and Plant Interactions in Pseudomonas syringae
Source: PLoS One. 2013 Mar 19;8(3):e59850. doi: 10.1371/journal.pone.0059850 (PMC3602303; doi:10.1371/journal.pone.0059850)
Supplement: Table S3 — Bacterial strains and plasmids. (DOCX) [file pone.0059850.s004.docx]

Table S3. Bacterial strains and plasmids

| **Strain or plasmid** | **Relevant characteristics**^a^ | **Source or reference** |
| --- | --- | --- |
| *E. coli* strains |  |  |
| TOP10 | F- *mcr*A Δ(*mrr-hsd*RMS-*mcr*BC) φ80*lac*ZΔM15 Δ*lac*X74 *rec*A1 *ara*D139 Δ(*araleu*) 7697 *gal*U *gal*K *rps*L (StrR) *end*A1 *nup*G | Invitrogen (Carlsbad, CA) |
| S17-1 | *recA* RP4-2-Tc::Mu-Km::Tn7 | (58) |
| *P. syringae* strains |  |  |
| *P. syringae* B728a | Wild type, Rf^R^, NFT^R^ | (56) |
| Δ*flgK* | *P. syringae* B728a with a *flgK* deletion, Rf^R^,NFT^R^, Km^R^ | A. Burch (unpublished) |
| Δ*flgM* | *P. syringae* B728a with a *flgM* deletion, Rf^R^, NFT^R^ | This work |
| Δ*gacS* | *P. syringae* B728a with a *gacS* deletion, Rf^R^, NFT^R^ | D. Gross (unpublished) |
| Δ*salA* | *P. syringae* B728a with a *salA* deletion, Rf^R^, NFT^R^ | D. Gross (unpublished) |
| Plasmids |  |  |
| p519nGFP | Broad host range vector, GFP^+^, Km^R^ | (69) |
| p519empty | p519nGFP with GFP removed | This work |
| p519Mcomp | p519n harboring the *flgM* complementing cassette | This work |
| pFLP2Ω | bhr vector expressing the Flp-recombinase, Sp^R^ | (59) |
| pTOK2T | Mobilizable, suicide vector, Tc^R^ | (27) |
| p*flgM*-KO | pTOK2T harboring the *flgM*-knock out cassette, Km^R^, Tc^R^ | This work |
| pKD13 | Source of kan-FRT fragment | (66) |
| pP*_fliC-gfp_* | pPROBE-GT harboring the *fliC* promoter, Gm^R^ | (59) |
| pP*_syfA-gfp_* | pPROBE-OT harboring the *syfA* promoter, Sp^R^ | (20) |

^a^ Rf, rifampicin; Gm, gentimicin; Sp, spectinomycin; Km, kanamycin; Tc, tetracycline; NFT, nitrofurantoin
