# Supplementary material for: Thermo-Regulation of Genes Mediating Motility and Plant Interactions in Pseudomonas syringae
Source: PLoS One. 2013 Mar 19;8(3):e59850. doi: 10.1371/journal.pone.0059850 (PMC3602303; doi:10.1371/journal.pone.0059850)
Supplement: Table S4 — Primers used in cloning and qRT-PCR. (DOCX) [file pone.0059850.s005.docx]

Table S4. Primers used in cloning and qRT-PCR

| **Primer** | **Sequence** |
| --- | --- |
| 5'-S-*flgM* | 5'-GACCCACCAGCTGAGTTC-3' |
| 5'-AS-*flgM*^a^ | 5'-GAAGCAGCTCCAGCCTACACACATGATTGAAAAAACCTCTGG-3' |
| 3'-S-*flgM*^a^ | 5'-GGTCGACGGATCCCCGGAATAACGCTAAGCCAAGGC-3' |
| 3'-AS-*flgM* | 5'-CGTCGACCAGTTGCG-3' |
| pKD4/13-site-1-S | 5'-GTGTAGGCTGGAGCTGCTTC-3' |
| pKD13-site-4-AS | 5'-ATTCCGGGGATCCGTCGACC |
| *flgM*-coding-S | 5'-CCAGAGGTTTTTTCAATCATG-3' |
| *flgM*-coding-AS | 5'-CAGCCTTGGCTTAGCG-3' |
| *flgM*-5'-S-complement | 5'-ATCGTCTAGACGTCACCGAAGAAGACG-3' |
| *flgM*-3'-AS-complement | 5'-ATCGTCTAGACTAGCGTTGGGTTTCG-3' |
| flgKe-xhoF | 5'-AGTCTAGAACGCCGCGGAAGGTGAACGTC-3' |
| flgKe-xbaR | 5'-AGCTCGAGGGAATCGTGAGCCATGCGTATC-3' |
| flgKs-hindF | 5'- TGAAGCTTTGCAACGGCCGCCACTGACG-3' |
| flgKs-speR | 5'- ACACTAGTGAGTCAAACCTTATAAAGTCGTGGAAG-3' |
| **qRT-PCR primers** |  |
| *rpoD*-RT-S | 5'-ACGCGCCATCATGCAGCTGTG-3' |
| *rpoD*-RT-AS | 5'-GCCAGTGCGTCAGTCCAGCTTTC-3' |
| 3981-RT-S | 5'-CAGCGGACGGGTGCAGGTAGAC-3' |
| 3981-RT-AS | 5'-CGTCGTTGTCAGGCTCGTCCAG-3' |
| *fliC*-RT-S | 5'-CGGCGCCTCGAACCAGATCTC-3' |
| *fliC*-RT-AS | 5'-CGCGGCGATTGAAGCAGAGAAG-3' |
| *syfA*-RT-S | 5'-CGGCCCGACTGAAACCACTGTG-3' |
| *syfA*-RT-AS | 5'-CCCCGCACCACCGATGTACAAC-3' |

^a^ Underlined sequence is complementary to kan-FRT from pKD13
